# Clinical genetic testing in four highly suspected pediatric restrictive cardiomyopathy cases

**DOI:** 10.1186/s12872-022-02675-w

**Published:** 2022-05-25

**Authors:** Min Zheng, Hong Huang, Xu Zhu, Harvey Ho, Liling Li, Xiaojuan Ji

**Affiliations:** 1 Children’s Hospital of Chongqing Medical University, National Clinical Research Center for Child Health and Disorders, Ministry of Education Key Laboratory of Child Development and Disorders, China International Science and Technology Cooperation Base of Child Development and Critical Disorders, Chongqing Key Laboratory of Child Infection and Immunity, 136 Zhongshan 2nd Road, Yu Zhong District, Chongqing, 400014 China; 2 Pediatric Department, North-Kuanren General Hospital of Chongqing, Chongqing, 401121 China; 3 Auckland Bioengineering Institute, The University of Auckland, Private Bag 92019, Auckland, 1142 New Zealand

**Keywords:** Restrictive cardiomyopathy, TNNI3, Mutation, Constrictive pericarditis

## Abstract

**Background:**

Restrictive cardiomyopathy (RCM) presents a high risk for sudden cardiac death in pediatric patients. Constrictive pericarditis (CP) exhibits a similar clinical presentation to RCM and requires differential diagnosis. While mutations of genes that encode sarcomeric and cytoskeletal proteins may lead to RCM, infection, rather than gene mutation, is the main cause of CP. Genetic testing may be helpful in the clinical diagnosis of RCM.

**Methods:**

In this case series study, we screened for *TNNI3*, *TNNT2*, and *DES* gene mutations that are known to be etiologically linked to RCM in four pediatric patients with suspected RCM.

**Results:**

We identified one novel heterozygous mutation, c.517C>T (substitution, position 517 C → T) (amino acid conversion, p.Leu173Phe), and two already known heterozygous mutations, c.508C>T (substitution, position 508, C → T) (amino acid conversion, p.Arg170Trp) and c.575G>A (substitution, position 575, G → A) (amino acid conversion, p.Arg192His), in the *TNNI3* gene in three of the four patients.

**Conclusion:**

Our findings support the notion that genetic testing may be helpful in the clinical diagnosis of RCM.

## Background

Restrictive cardiomyopathy (RCM) is a rare cardiomyopathy in which the cardiac walls are rigid and the heart is restricted from stretching and filling properly, thus presenting a high risk for cardiac death in pediatric patients [1]. To diagnose RCM, it is crucial to rule out constrictive pericarditis (CP), another cardiac disorder that has similar clinical presentations and imaging manifestations but different pathophysiological alterations, prognoses, and treatments [2]. For example, in RCM, diastolic dysfunction is caused by abnormal elastic properties of the myocardium and/or intercellular matrix, whereas in CP, diastolic dysfunction is caused by external pericardial constraints [2]. The treatments for RCM and CP are also different. Currently, there are no curative treatments for RCM and the prognosis is generally very poor [3]. Cardiac transplantation is the only effective treatment [3], while CP can often be remedied surgically.

As mentioned above, differentiating RCM from CP in clinical diagnosis can be challenging [2], and many clinical cases have been diagnosed by thoracotomy, which is invasive and associated with high mortality [4]. There are a number of mutations in genes encoding sarcomeric and cytoskeletal proteins, including *TNNI3, ACTC, β-MHC, TNNT2, TNNC1, DES, MYH, MYL3, and CRYAB,* that have been reported to be etiologically linked to RCM [5]. These gene mutations are distinct from CP or infiltration of cardiac muscle that are usually caused by other pathogenic factors. Thus, it has been suggested that genetic screening of genes encoding sarcomeric proteins could be an important tool to clinically diagnose RCM [6].

In this case series study, we screened gene mutations in *TNNI3, TNNT2*, and *DES*, which have been shown to be pathogenic for RCM, in four pediatric patients with suspected RCM and whose transthoracic echocardiography (TTE) presented a similar restricted ventricular filling pattern.

## Methods

### Ethical approval

This study was approved by the Ethics Committee of Children’s Hospital of Chongqing Medical University (074/2013), all methods were carried out in accordance with relevant guidelines and regulations. Informed consent was obtained from the parents of the pediatric patients.

### Patients

Four pediatric patients with suspected RCM were admitted to our hospital and underwent TTE examination, which revealed typical signs of a restrictive filling pattern with abnormal E/A ratios and isovolumic relaxation times. The clinical data of these four patients, including family history, disease onset time, clinical symptoms, physical signs, and the results of TTE and other diagnostic information, were obtained from our hospital database. TTE was performed in accordance with the recommendations by the American Society of Echocardiography [7, 8].

### Variant analysis

Genomic DNA was extracted from peripheral blood samples using the Whole Blood DNA Mini kit (Yaneng BIO science Co., Ltd, Shenzhen, China) according to the manufacturer’s instructions. DNA concentration and purity were analyzed using the Nanodrop ND-2000 (Nanodrop Technologies Company, USA). Primer sequences used for polymerase chain reaction (PCR) in this study as follows: for DES8-9: 5′-TGTGCGATGGACCCTGTTAC-3′ (forward) and 5′-AGGCTCACTCACTGCCAACA-3′ (reverse); for TNNI3-7: 5′-CCAGGTTATGCCAGTGGTTTTG-3′ (forward) and 5′-CCCCTCAGCATCCTCTTTCC-3′ (reverse); for TNNI3-8: 5′-CTTAGGCATCCAGGGTAGAGT-3′ (forward) and 5′-GCAGTAGGCAGGAAGGC-3′ (reverse); for TNNT2-8: 5′-GGGGCAGTGCTGGAAGAT-3′ (forward) and 5′-GCAGTCAAGGAGCATCCAGTA-3′ (reverse); PCR products were analyzed using Sanger sequencing (a chain termination method) with the ABI 3730XL (Thermo Fisher Scientific Inc., Waltham, MA, USA) and further analyzed using the chromas 2.6.6 DNA sequencing Software (Technelysium Pty Ltd, South Brisbane, Australia).

Data from a control group that consisted of anonymous blood samples from 50 mixed-ancestry individuals of varying ages and genders were obtained from the clinical molecular biological laboratory.

Variants were named based on the nomenclature of the Human Genome Variation Society (http://www.hgvs.org/mutnomen). The reference sequences for nucleotides and amino acids were obtained from the National Center of Biotechnology Information (NCBI, http://www.ncbi.nlm.nih.gov). Protein IDs encoded by *TNNI3* were obtained from the UniProt Database (http://www.uniprot.org/).

## Results

### General information

Case 1: A 4-year, 5-month-old girl had clinical symptoms of fatigue and exertional shortness of breath. The concentration of serum B-type natriuretic peptide (BNP) was 2070 pg/ml. Abdominal ultrasonography revealed congestive hepatomegaly. Electrocardiogram (ECG) showed bi-atrial enlargement and diffuse ST-T wave changes. The girl eventually died. No family history of RCM or other cardiovascular diseases was reported.

Case 2: A 5-year-old girl had clinical symptoms of exertional fatigue, shortness of breath, and lower extremity edema. The concentration of serum BNP was 2243 pg/ml. Abdominal ultrasonography showed congestive hepatomegaly. ECG indicated ST-T wave changes. At age 6, the girl died suddenly at home. No family history of RCM or other cardiovascular diseases was reported.

Case 3: A 4-month, 21-day-old boy presented with clinical manifestations of cyanosis and late hoarseness. No auxiliary examination results were obtained except for echocardiography. The boy died of heart failure and multiple organ dysfunction soon after admission. No family history of RCM or other cardiovascular diseases was reported.

Case 4: A 7-year, 2-month-old girl was admitted to our hospital with symptoms of coughing and expectoration. The concentration of serum BNP was not obtained. Abdominal ultrasound examination showed congestive hepatomegaly and a large amount of peritoneal effusion. ECG revealed left atrial hypertrophy and a change in ST-T wave. At age 11, the girl died suddenly on her way to school. No family history of RCM or other cardiovascular diseases was reported.

### Imaging examination

**Fig. 1:**
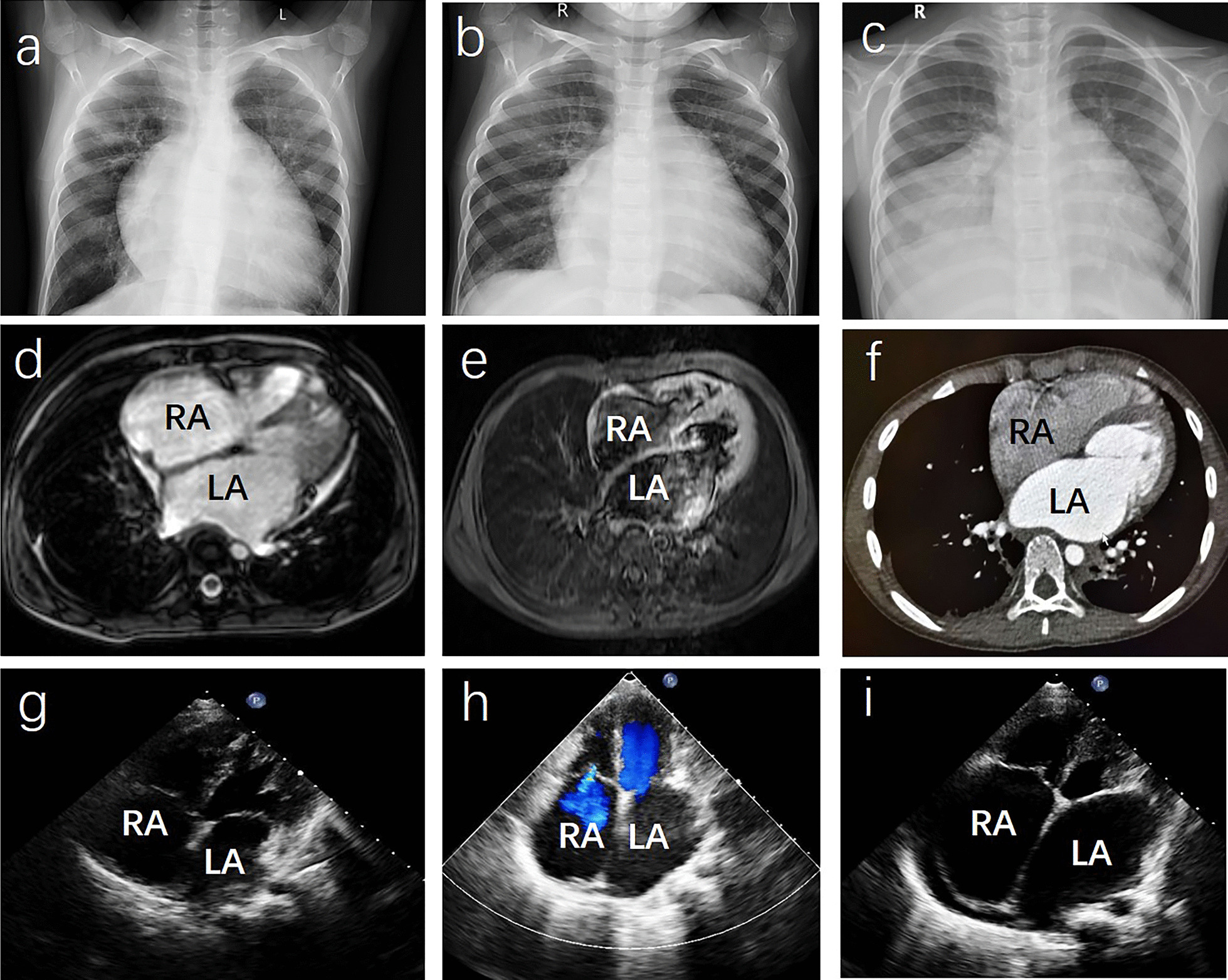
Representative images of cardiac X-ray, MRI, CTA, and two-dimensional echocardiography. *LA* left atrium, *RA* right atrium. **a** (case 1), **b** (case 2), **c** (case 4): Chest X-ray showed increased heart shadow without calcification of the pericardium, thickened pericardium, or endocardium. **d** (case 2), **e** (case 4): Cardiac MRI showed bi-atrial enlargement without calcification of the pericardium, thickened pericardium, or endocardium. **f** (case 4): Cardiac CTA showed bi-atrial enlargement without calcification of the pericardium, thickened pericardium, or endocardium. **g** (case 1), **h** (case 2), **i** (case4): Two-dimensional echocardiography showed dilation of the left and right atria with normal ventricular sizes, without calcification of the pericardium, thickened pericardium, or endocardium

**Fig. 2:**
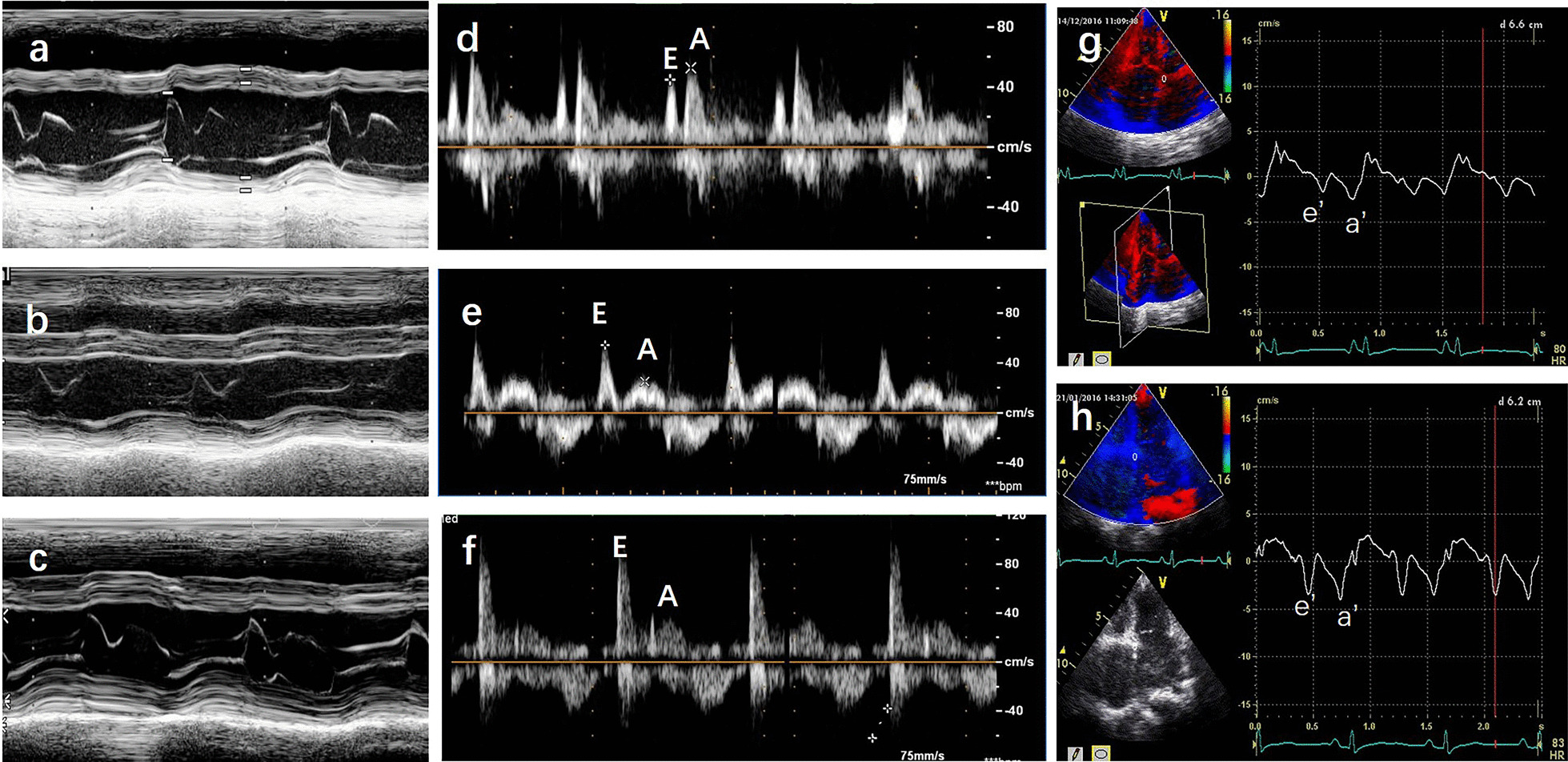
Representative images of M-mode echocardiography and diastolic function of left ventricles. E: peak mitral flow velocity in early diastole; A: peak mitral flow velocity in late diastole. IVRT: isovolumic relaxation time. E′: peak velocity of mitral annulus in early diastole; a′: peak velocity of mitral annulus in late diastole. **a** (case 1), **b** (case 2), **c** (case 4): M-mode echocardiography showed no abrupt septal movement (‘notch’ or ‘bounce’ in early diastole, or septal movement toward left ventricle in inspiration). **d** (case 1), **e** (case 2), **f** (case 4): Mitral flow velocity by pulsed Doppler echocardiography showing reduced cardiac diastolic function. **g** (case 1), **h** (case 4): Tissue velocity imaging of mitral annulus showing reduced cardiac diastolic function

Case 1: Chest X-ray showed increased heart shadow without calcification of the pericardium (Fig. 1a). Cardiac magnetic resonance imaging (MRI) revealed a bi-atrial enlargement without calcification of the pericardium, thickened pericardium, or endocardium (data not shown). Two-dimensional echocardiography showed that the left and right atria were enlarged, the right ventricle was slightly enlarged, and the left ventricle was normal in size, without calcification of the pericardium, thickened pericardium, or endocardium (Fig. 1g). M-mode echocardiography showed that the left ventricular ejection fraction was less than 55%, and there was no abrupt septal movement (‘notch’ or ‘bounce’ in early diastole, or septal movement toward left ventricle in inspiration) (Fig. 2a). Pulsed Doppler echocardiography showed that the ratio of peak E to peak A velocity of mitral valve flow was less than 1 (E/A < 1) and the isovolumic relaxation time (IVRT) was longer than 80 ms (Fig. 2d). Tissue velocity imaging showed that the peak e′ and peak a′ velocity of mitral annulus decreased with an e′/a′ < 1 and the peak e′ was < 8 cm/s (Fig. 2g).

Case 2: Chest X-ray showed increased heart shadow without calcification of the pericardium (Fig. 1b). Cardiac MRI revealed a bi-atrial enlargement without calcification of the pericardium, thickened pericardium, or endocardium (Fig. 1d). Two-dimensional echocardiography showed dilation of the left and right atria with normal ventricular sizes, without calcification of the pericardium, thickened pericardium, or endocardium (Fig. 1h). M-mode echocardiography showed that the ventricular septum and left ventricular wall were slightly thickened and the left ventricular ejection fraction was less than 55%, and there was no abrupt septal movement (‘notch’ or ‘bounce’ in early diastole, or septal movement toward left ventricle in inspiration) (Fig. 2b). Pulsed Doppler echocardiography showed that the peak E and peak A velocity of mitral valve flow decreased significantly (E/A > 2) and the IVRT was longer than 80 ms (Fig. 2e). Unfortunately, tissue velocity images were not obtained for this case.

Case 3: No auxiliary examination results were obtained except for echocardiography. Two-dimensional echocardiography showed dilation of the left and right atria with normal ventricular sizes, without calcification of the pericardium, thickened pericardium, or endocardium. M-mode echocardiography showed that the left ventricular ejection fraction was less than 55%, and there was no abrupt septal movement (‘notch’ or ‘bounce’ in early diastole, or septal movement toward left ventricle in inspiration). Pulsed Doppler echocardiography showed that the ratio of peak E to peak A velocity of mitral valve flow was more than 2 (E/A > 2) and the IVRT was longer than 80 ms. Unfortunately, tissue velocity images were not obtained and the TTE images of this case were lost.

Case 4: Chest X-ray showed increased heart shadow without calcification of the pericardium (Fig. 1c). Cardiac MRI (Fig. 1e) and cardiac computer tomography angiography (CTA) (Fig. 1f) showed bi-atrial enlargement without calcification of the the pericardium, thickened pericardium, or endocardium. Two-dimensional echocardiography showed dilation of the left and right atria with normal ventricular sizes, without calcification of the pericardium, thickened pericardium, or endocardium. (Fig. 1i).M-mode echocardiography showed that the left ventricular ejection fraction was greater than 55%, and there was no abrupt septal movement (‘notch’ or ‘bounce’ in early diastole, or septal movement toward left ventricle in inspiration) (Fig. 2c). Pulsed Doppler echocardiography showed that the ratio of peak E to peak A velocity of mitral valve flow was more than 2 (E/A > 2) and the IVRT was longer than 80 ms (Fig. 2f). Tissue velocity imaging showed that the peak e′ and peak a′ velocity of mitral annulus decreased with an e′/a′ < 1, and the peak e′ was < 8 cm/s (Fig. 2h).

### *TNNI3* mutations

**Fig. 3:**
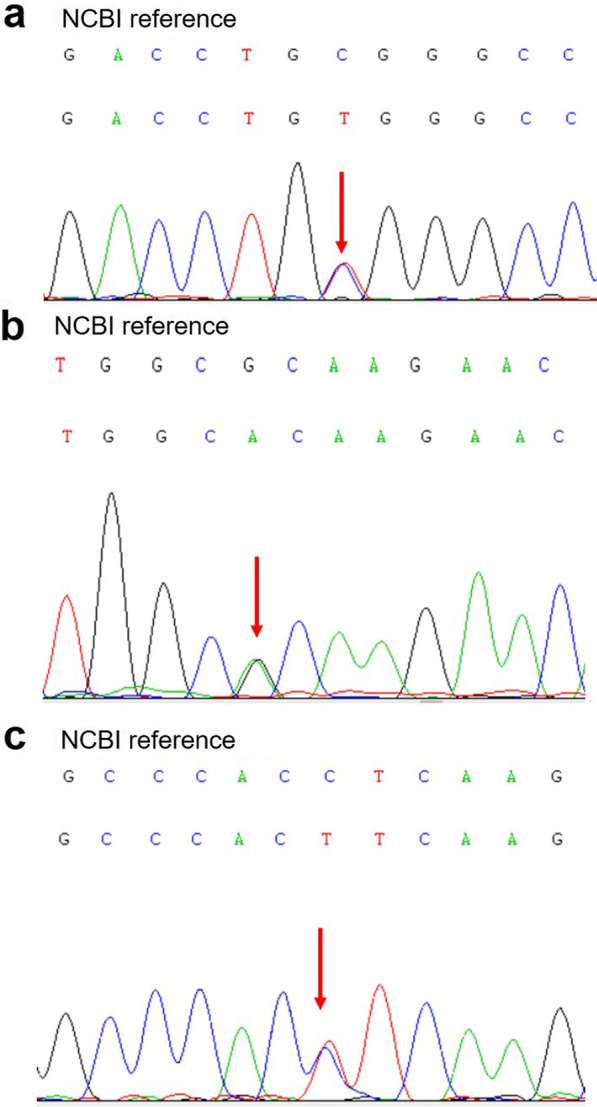
Sanger sequencing validation of the mutation sites. **a** *TNNI3* gene mutation at position 508 (c.508C>T) in case 1. **b** *TNNI3* gene mutation at position 575 (c.575G>A) in case 2. **c** *TNNI3* gene mutation at position 517 (c.517C>T) in case 3

Sanger sequencing using double orientation primers identified three *TNNI3* mutations in cases 1, 2, and 3, including one novel and two already known mutations (Fig. 3). These missense mutations included a heterozygous mutation at the nucleotide position 508 (c.508C>T) in case 1, resulting in the substitution of arginine with tryptophan at amino acid position 170 (p.Arg170Trp), a heterozygous mutation at position 575 (c.575G>A) in case 2, resulting in the substitution of arginine with histidine at amino acid position 192 (p.Arg192His), and a novel heterozygous mutation at position 517 (c.517C>T) in case 3, resulting in the substitution of leucine with phenylalanine at amino acid position 173 (p.Leu173Phe). No mutations were identified in case 4, the parents and the elder sister of case 1, or the control group.

All these mutations were not found in 1000Genomes, ESP and EXAC databases, but were revealed by REVEL, SIFT, Polyphen2 and Mutation Taster prediction tools to have a high probability of damaging effect. c.508C>T and c.575G>A were reported in ClinVar in association with cardiomyopathy. Thus, according to ACMG guidelines [9], c.508C>T and c.575G>A were classified as pathogenic variants, while c.517C>T was classified as an uncertain significance variant.

### *TNNT2* and *DES* mutations

No mutations were found in *TNNT2* and *DES* in the four patients or in the control group.

## Discussion

RCM is a myocardial disorder characterized by elevated myocardial stiffness that leads to restrictive diastolic dysfunction. RCM is also associated with limited unilateral or bilateral ventricular filling resulting from myocardial interstitial fibrous hyperplasia from cardiomyopathy that decreases diastolic volume [10]. RCM patients present with variable clinical manifestations. Patients with end-stage right heart failure are mainly characterized by systemic blood stasis (such as in the jugular vein), hepatomegaly, ascites, lower limb edema, and increased venous pressure [11, 12], whereas some patients may have symptoms linked to left heart failure such as dyspnea, hemoptysis and wet rales at the bottom of the lung, low cardiac output, syncope, and even thromboembolism or sudden death [13]. Other non-specific manifestations of RCM include fatigue, shortness of breath, impaired activity tolerance, and slow physical development [11–13].

RCM may be diagnosed in accordance with the following criteria: 1) a restrictive left ventricular filling pattern in the absence of obviously known causes, and 2) exclusion of CP. A differential diagnosis between RCM and CP may be made by medical history, as well as physical and auxiliary examinations including TTE, chest X-ray, MRI, cardiac catheterization, myocardial biopsy, and blood tests. The main indicators used for differential diagnosis of RCM and CP are shown in Table 1 [14–18].

**Table 1:** Major indicators for differential diagnosis between RCM and CP

	RCM	CP
Pericardial calcification (TTE, Chest X-ray, CT, MRI)	Rare	+
Thickened pericardium (TTE, Chest X-ray, CT, MRI)	0	+
Thickened endocardium (TTE, CT, MRI)	Sometimes	0
Abrupt septal movement (‘notch’ or ‘bounce’) in early diastole (TTE)	0	+
Septal movement toward left ventricle in inspiration (TTE)	0	+
Left and right atrial enlargement (TTE, CT, MRI)	+	+
the tissue velocity of mitral annulus (TTE)	< 8 cm/s	> 8 cm/s
systolic area index (Cardiac catheterization)	0.92 ± 0.19	1.4 ± 0.2
End-diastolic pressure difference between left and right ventricles (Cardiac catheterization)	> 5 mmHg	< 5 mmHg
the ratio of right ventricular end-diastolic pressure to systolic pressure (Cardiac catheterization)	0.35 ± 0.14	0.50 ± 0.13
Radionuclide retention in atrium, Delayed radionuclide imaging of right ventricle (Radionuclide examination)	+	0
myocardial interstitial fibrosis (Endomyocardial biopsy)	+	Rare
B-type natriuretic peptide	Can be higher than 800 pg/ml	Most between 100 ~ 200 pg/ml

*RCM* restrictive cardiomyopathy, *CP* constrictive pericarditis, *CT* computed tomography, *MRI* magnetic resonance imaging, *TTE* transthoracic echocardiography

However, it should be noted that distinguishing CP from RCM diagnosis according to the abovementioned diagnostic criteria can be challenging. In difficult cases, genetic testing may be considered when cardiologists have established a clinical index of suspected RCM based on a patient’s clinical records, family history, and electrocardiographic/echocardiographic phenotypes. Indeed, a recent genetic study on pediatric RCM patients suggested that 75% of RCM patients exhibited genetic mutations [19]. Genetic testing is not only important in differentiation between RCM and CP as mentioned above, but also critical in obtaining the final diagnosis of other cardiac diseases such as hypertrophic cardiomyopathy, inherited heart diseases in athletes [20], and sudden cardiac death [21, 22].

Mutations of a number of genes encoding for sarcomeric and cytoskeletal proteins are associated with the etiology of RCM with an incident rate of 33–66% (Table 2) [1, 6, 11, 23–35]. *TNNI3* was the first sarcomere gene reported to be pathogenic for RCM when mutated [11]. In contrast, CP is a disease of the pericardium resulting from chronic inflammation and/or scarring, and familiar CP is extremely rare. A homozygous deletion mutation in exon 6 of the proteoglycan 4 gene (*PRG4*), c.884_885delAG, was reported to be etiologically correlated to familiar CP [10], but this remains to be further corroborated.

**Table 2:** Genetic mutations associated with RCM

Gene loci	Gene name
ACTC1 [29]	α-Cardiac actin
BAG3 [30]	BCL2-associated athanogene 3
CRYAB [31]	αB-crystallin
DES [32]	Desmin
GLA	α-Galactosidase
MYH7 [11]	β-Myosin heavy chain 7
MYL2	Myosin regulatory light chain 2,slow
MYL3	Myosin light chain 3, slow
MYPN [35]	Myopalladin
TNNI3 [1, 6, 34]	Cardiac troponin I, type 3
TNNT2 [1]	Cardiac troponin T, type 2
TPM1	α-Tropomyosin 1
TTN [35]	Titin
TTR	Transthyretin
MYBPC3 [23]	cMyBP-C
FLNC [24, 25]	filamin C
MYPN [26]	myopalladin
LMNA [28]	Lamin A
ABCC9 [27]	Sur2A

In the present study, we genetically investigated four pediatric cases whose TTE revealed a similar RCM pattern, including bi-atrial enlargement with normal ventricular chamber sizes and abnormal diastolic functions. We identified three missense mutations in the *TNNI3* gene: p.Arg170Trp (case 1), p.Arg192His (case 2), and p.Leu173Phe (case 3). The first two mutations have been reported to be etiologically linked to RCM [36–39], but the p.Leu173Phe mutation was novel. Although it is currently unclear whether this novel p.Leu173Phe mutation is pathogenic for RCM, we noted that a number of missense mutations around Leu173, such as p.R170R, p.A171T, and p.K178E, have been reported to cause RCM [40]. Hence, we propose that this novel p.Leu173Phe mutation is pathogenic for RCM, although functional studies are warranted to verify our hypothesis in the future.

Mechanistically, *TNNI3* mutations are associated with impaired diastolic functions due to myofibril Ca^2+^ hypersensitivity [12, 41], thus negatively affecting the actin–troponin–tropomyosin complex interaction [42]. It has been hypothesized that changes in actin-binding affinity to troponin C, and the ability to inhibit thin filaments during diastole caused by certain *TNNI3* mutations, lead to an altered interaction within the actin–troponin–tropomyosin complex, causing severe diastolic dysfunction [42]. Hence, the *TNNI3* mutations found in this case series study may cause reduced cardiac diastolic function. Therefore, the children carrying *TNNI3* missense mutations, combined with their clinical history and results from auxiliary examinations, could have been diagnosed with RCM.

## Conclusion

We report here that *TNNI3* mutations, including one novel missense mutation, p.Leu173Phe, and two already known mutations, p.Arg170Trp and p.Arg192His, were identified in three of four highly suspected pediatric RCM cases. Our findings contribute to the knowledge about the genetic basis of RCM and support the notion that genetic testing could be helpful in the clinical diagnosis of RCM, especially when exclusion of CP is difficult.

## Data Availability

The data that support the findings of this study are available from the corresponding author upon reasonable request.

## Abbreviations

RCM: Restrictive cardiomyopathy
CP: Constrictive pericarditis
TTE: Transthoracic echocardiography
ECG: Electrocardiogram
BNP: B-type natriuretic peptide
MRI: Magnetic resonance imaging
CTA: Tomography angiography
IVRT: Isovolumic relaxation time
E/A: The ratio of peak E to peak A velocity of mitral valve flow
